# The Association of Pre-existing Diagnoses of Alzheimer’s Disease and Parkinson’s Disease and Coronavirus Disease 2019 Infection, Severity and Mortality: Results From the Korean National Health Insurance Database

**DOI:** 10.3389/fnagi.2022.821235

**Published:** 2022-03-03

**Authors:** Ji Hee Kim, In Bok Chang, Yoo Hwan Kim, Chan Yang Min, Dae Myoung Yoo, Hyo Geun Choi

**Affiliations:** ^1^Department of Neurosurgery, Hallym University College of Medicine, Anyang, South Korea; ^2^Department of Neurology, Hallym University College of Medicine, Anyang, South Korea; ^3^Hallym Data Science Laboratory, Hallym University College of Medicine, Anyang, South Korea; ^4^Department of Otorhinolaryngology-Head and Neck Surgery, Hallym University College of Medicine, Anyang, South Korea

**Keywords:** Alzheimer’s disease, Coronavirus disease 2019 (COVID-19), dementia, neurodegeneration, neurodegenerative disease, Parkinson’s disease

## Abstract

**Objectives:**

Despite the numerous studies on coronavirus disease 2019 (COVID-19), data regarding the impact of pre-existing diagnoses of Alzheimer’s disease (AD) and Parkinson’s disease (PD) on the susceptibility to and outcome of COVID-19 are limited. We aimed to determine whether patients with AD/PD had a higher likelihood of contracting COVID-19 and experiencing worse outcomes.

**Methods:**

Data from patients with confirmed diagnoses of COVID-19 (*n* = 8,070) from January to June 2020 and control participants (*n* = 121,050) who were randomly selected to match the patients on the basis of age and sex were extracted from the Korean National Health Insurance Database. Pre-existing diagnoses of AD and PD were identified based on medical claim codes. The associations of pre-existing AD or PD with contracting COVID-19, developing severe COVID-19 and dying due to COVID-19 were examined using a logistic regression model. The participants’ age, sex, income, comorbidity score, and history of hypertension/diabetes were assessed as covariates.

**Results:**

COVID-19 cases were more likely to have a pre-existing AD diagnosis (adjusted odds ratio [aOR] = 2.11, 95% confidence interval [CI] = 1.79–2.50, *P*-value < 0.001) than controls. COVID-19 cases were more likely to have a pre-existing PD diagnosis than controls, although this estimate did not quite reach statistical significance (aOR = 1.41, 95% CI = 1.00–2.00, *P*-value = 0.054). Pre-existing AD was related to severe disease and mortality from COVID-19 (aOR = 2.21, 95% CI = 1.64–2.98; aOR = 2.21, 95% CI = 1.00–2.00). Pre-existing PD was not associated with mortality (aOR = 1.54, 95% CI = 0.75–3.16) but was associated with severe disease (aOR = 2.89, 95% CI = 1.56–5.35).

**Conclusion:**

We found that COVID-19 infection was significantly associated with a pre-existing diagnosis of AD but not with a pre-existing diagnosis of PD. Patients with pre-existing AD had higher odds of developing severe COVID-19 and dying. Pre-existing PD was only associated with a higher odds of developing severe COVID-19.

## Introduction

Coronavirus disease 2019 (COVID-19), which is caused by severe acute respiratory syndrome coronavirus-2 (SARS-CoV-2), has affected 224 countries, with more than 151,000,000 confirmed cases globally (World Health Oroganizations [WHO], 2021). This disease is primarily a respiratory disease that ranges in severity from mild to fatal, but it also impacts the functioning of the cardiovascular, renal, and nervous systems (Yuki et al., 2020). Although all individuals are susceptible to infection by SARS-CoV-2, older individuals and those with chronic diseases, specifically hypertension, coronary artery disease, obesity, and diabetes, are more susceptible to infection by SARS-CoV-2, the development of severe clinical symptoms of COVID-19 and mortality due to COVID-19 (Alzheimer’s and Dementia, 2020). Furthermore, there is growing concern that the chronic diseases that make individuals more vulnerable to contracting COVID-19 and developing adverse outcomes could include neurodegenerative diseases. One recently published meta-analysis highlighted an imperative role of mental and neurological disorders in the context of COVID-19 and suggested instructions for recognizing and protecting these vulnerable individuals in the pandemic (Liu et al., 2021).

Neurodegenerative diseases are a heterogeneous group of diseases that are characterized by the progressive loss of neuronal cells of the central or peripheral nervous systems, including vascular dementia, Alzheimer’s disease (AD), Parkinson’s disease (PD), frontotemporal dementia (FTD), and various tauopathies (Yu et al., 2021). AD is the most common neurodegenerative disorder and the most frequent cause of dementia and is characterized by a progressive deterioration in cognition, particularly in memory function. PD is the most common movement disorder and represents the second most common degenerative disease of the central nervous system. The number of people suffering from these neurodegenerative disorders increases with age, and these disorders are often accompanied by various comorbidities. Therefore, it is plausible that patients affected by these neurodegenerative disorders would more likely be susceptible to SARS-CoV-2 infection. In addition, it is necessary to investigate whether people living with AD or PD are at greater risk of a severe clinical course and unfavorable outcomes of COVID-19. However, documented reports of the impacts of neurodegenerative disease on COVID-19 remain scarce. A recent cohort study used the UK Biobank to examine the associations of several risk factors, including all-cause dementia, AD and PD in particular, with COVID-19 positivity, severity (hospitalization), and death (Tahira et al., 2021). Another cohort study comprising 363 patients with AD and 259 patients with PD selected from a sample of 3,732 individuals concluded that inpatients with AD have a higher risk of 28-day mortality from COVID-19 (Fathi et al., 2021). In addition, an observational case series study investigating the frequency and mortality of COVID-19 among patients with a previous diagnosis of AD and FTD suggested that living in care homes was the most relevant factor for a higher risk of COVID-19 infection and death, with AD patients having a greater risk than those with FTD (Matias-Guiu et al., 2020).

We thus aimed to examine the associations between pre-existing AD and PD and COVID-19 infection, severity, and mortality using the Korean National Health Insurance Database.

## Materials and Methods

### Ethics

Approval for this study was obtained from the ethics committee of Hallym University (2020-07-022). The need to obtain written informed consent was waived by the Institutional Review Board.

### Study Population and Participant Selection

We used data extracted from the Korea National Health Insurance Database for Coronavirus disease 2019 (NHID-COVID DB). The NHID-COVID DB provided the data of all individuals who underwent testing for SARS-CoV-2 infection with real-time reverse-transcriptase-polymerase chain reaction (RT-PCR) assays of nasal or pharyngeal swabs in accordance with the World Health Organization (WHO) guidelines. The data covers the entire country without any exception based on medical claim codes between 2015 and 2020, including demographics, treatment outcomes, the isolation period, and the confirmation date of COVID-19 infection by PCR. In addition, all Korean citizens are registered with a lifelong 13-digit resident registration number and are required to register in the National Health Insurance Service (NHIS). Since the 13-digit resident registration number is used in all hospitals and clinics in Korea, all medical records of the entire population can be tracked and overlapping duplication can be prevented.

**FIGURE 1:**
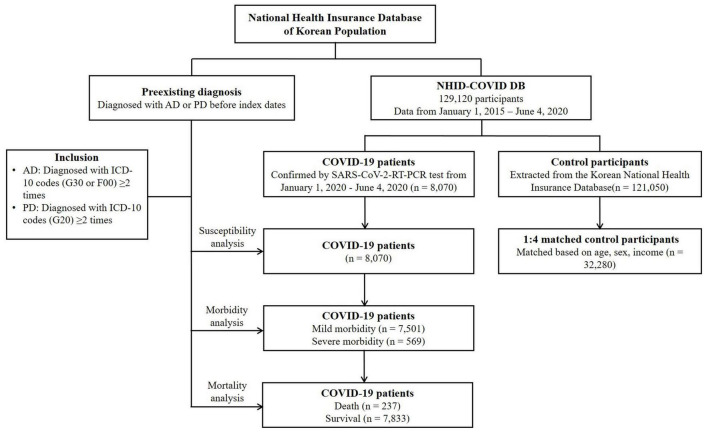
A flowchart of the screening process used in this study. From a total of 129,120 participants, 8,070 COVID-19 patients were matched for age, sex, and income with 32,280 control participants. AD, Alzheimer’s disease; ICD-10, International Classification of Diseases, Tenth Revision; NHID-COVID DB, National Health Insurance Database Coronavirus disease 2019; PD, Parkinson’s disease; RT-PCR, reverse-transcriptase-polymerase chain reaction.

Data from patients with confirmed cases of COVID-19 were collected from 1 January 2020 to 4 June 2020. All included patients terminated treatment or died before 4 June 2020 (*n* = 8,070). The control participants were proportionally sampled in a 15:1 ratio with the COVID-19 patients from the Korean National Health Insurance Database after stratification by age and sex (*n* = 121,050). Then, the COVID-19 patients and control participants were matched at a ratio of 1:4 based on age, sex, and income. Control participants without income records were excluded (*n* = 2,136). To mitigate potential selection bias, control participants were randomly chosen using clustered sampling. The index date was determined as the date of the confirmation of the diagnosis of COVID-19. The index date for each control participant was randomly assigned from 1 January 2020 to 4 June 2020. Ultimately, 8,070 COVID-19 patients were matched with 32,280 control participants (Figure 1).

### Exposure (Alzheimer’s Disease/Parkinson’s Disease)

Participants were considered to have AD if they were diagnosed with AD [International Classification of Diseases and Related Health Problems (ICD-10) code: G30] or dementia in AD (F00). Patients with PD were those who had been diagnosed with PD (ICD-10 code: G20). To ensure the accuracy of the diagnosis, only participants who had received treatment ≥2 times were included, as in our previous work (Choi et al., 2019; Kim et al., 2019).

### Primary Outcome (Coronavirus Disease 2019 Infection)

A confirmed diagnosis of COVID-19 was based on a positive assay for SARS-CoV-2.

### Secondary Outcomes (Coronavirus Disease 2019 Severity and Mortality)

The severity of COVID-19 was divided into mild (*n* = 7,501) and severe (*n* = 569). Severe COVID-19 was defined based on admission to the intensive care unit (ICU), the use of invasive ventilation or extracorporeal membrane oxygenation (ECMO), and mortality. Additionally, COVID-19 patients were classified as having died (*n* = 237) or survived (*n* = 7,833).

### Covariates

There were nine age groups at 10-year intervals: 0–9, 10–19, 20–29…, and 80+ years old. Income groups were classified as low, middle, and high. Individual comorbid conditions in this study were assessed according to the Charlson Comorbidity Index (CCI), which assesses 17 comorbidities and is presented as a continuous score from 0 to 29 (Quan et al., 2011), excluding dementia and diabetes.

### Statistical Analysis

Differences in demographic data and general characteristics between the COVID-19 group and the control group and between the mild COVID-19 group and severe COVID-19 group were examined using the chi-square test or Fisher’s exact test, as appropriate.

To estimate the odds of COVID-19 infection, odds ratios (ORs) with 95% confidence intervals (CIs) in patients with pre-existing AD or PD were calculated using crude (simple model) and adjusted conditional logistic regression models (adjusted for CCI score, hypertension, and diabetes).

To estimate the risks of severe COVID-19 and mortality in patients with pre-existing AD or PD, unmatched analyses were performed using unconditional logistic regression.

Stratified analyses were performed with subgroups of patients with different ages (<50 years old and ≥50 years old), sexes, incomes (low, middle, and high), CCI scores (0 points, 1 point, and ≥2 points), histories of hypertension, and histories of diabetes to test whether the effect of pre-existing AD or PD was consistent across different groups.

We used SAS version 9.4 (SAS Institute Inc., Cary, NC, United States) for the statistical analysis. All statistical tests were two-tailed, and probability values less than 0.05 were regarded as significant.

## Results

The distributions of age, sex, and income were the same between the COVID-19 and control groups owing to the matching process (*P* = 1.000). The COVID-19 group had higher proportions of individuals with a CCI score of 2 or higher, diabetes, AD, and PD than the control group (each *P* < 0.001). In the COVID-19 group, compared to patients with mild disease, the group of patients with severe disease was older and had a higher proportion of men. In addition, patients with severe COVID-19 were more likely to have a high income, a CCI score of 2 or higher, hypertension, diabetes, AD, and PD (each *P* < 0.001). The demographic and clinical characteristics of these cohorts are summarized in Table 1.

**TABLE 1:** General characteristics of the participants.

Characteristics	Total participants			COVID-19 patients		
	COVID-19 (n, %)	Control (n, %)	*P*-value	Severe morbidity (n, %)	Mild morbidity (n, %)	*P*-value
Total number	8,070 (100.0)	32,280 (100.0)		569 (100.0)	7,501 (100.0)	
Age (years)			1.000			<0.001*
0–9	81 (1.0)	324 (1.0)		6 (1.1)	75 (1.0)	
10–19	276 (3.4)	1,104 (3.4)		6 (1.1)	270 (3.6)	
20–29	2,057 (25.5)	8,228 (25.5)		31 (5.5)	2,026 (27.0)	
30–39	832 (10.3)	3,328 (10.3)		25 (4.4)	807 (10.8)	
40–49	1,036 (12.8)	4,144 (12.8)		30 (5.3)	1,006 (13.4)	
50–59	1,567 (19.4)	6,268 (19.4)		71 (12.5)	1,496 (19.9)	
60–69	1,199 (14.9)	4,796 (14.9)		116 (20.4)	1,083 (14.4)	
70–79	617 (7.7)	2,468 (7.7)		118 (20.7)	499 (6.7)	
80+	405 (5.0)	1,620 (5.0)		166 (29.2)	239 (3.2)	
Sex			1.000			<0.001*
Men	3,236 (40.1)	12,944 (40.1)		306 (53.8)	2,930 (39.1)	
Women	4,834 (59.9)	19,336 (59.9)		263 (46.2)	4,571 (60.9)	
Income			1.000			<0.001*
1 (low)	3,105 (38.5)	12,420 (38.5)		196 (34.5)	2,909 (38.8)	
2	2,347 (29.1)	9,388 (29.1)		161 (28.3)	2,186 (29.1)	
3 (high)	2,618 (32.4)	10,472 (32.4)		212 (37.3)	2,406 (32.1)	
CCI score^†^			<0.001*			<0.001*
0	6,725 (83.3)	29,637 (91.8)		333 (58.5)	6,392 (85.2)	
1	869 (10.8)	1,514 (4.7)		110 (19.3)	759 (10.1)	
≥2	476 (5.9)	1,129 (3.5)		126 (22.1)	350 (4.7)	
Hypertension	1,657 (20.5)	6,535 (20.2)	0.331	275 (48.3)	1,382 (18.4)	<0.001*
Diabetes	969 (12.0)	3,406 (10.6)	<0.001*	186 (32.7)	783 (10.4)	<0.001*
Alzheimer’s disease	309 (3.8)	647 (2.0)	<0.001*	117 (20.6)	192 (2.6)	<0.001*
Parkinson’s disease	52 (0.6)	109 (0.3)	<0.001*	275 (48.3)	1,382 (18.4)	<0.001*

*CCI, Charlson comorbidity index; COVID-19, Coronavirus Disease 2019.*

*Chi-squared or Fisher’s exact test. Significance at p < 0.05.

*^†^CCI score was assessed excluding dementia and diabetes.*

The crude and adjusted ORs of the association between pre-existing AD or PD and COVID-19 are shown in Table 2. COVID-19 cases were more likely to have a pre-existing diagnosis of AD than those of controls (adjusted OR in model 2 = 2.11, 95% CI = 1.79–2.50, *P* < 0.001). COVID-19 cases were more likely to have a pre-existing diagnosis of PD than those of controls, although the estimates did not reach statistical significance (adjusted OR in model 2 = 1.41, 95% CI = 1.00–2.00, *P* = 0.054).

**TABLE 2:** Crude and adjusted odds ratios of the association between pre-existing Alzheimer’s disease or Parkinson’s disease and COVID-19 in the total study participants.

Characteristics	COVID-19 (exposure/total, %)	Control (exposure/total, %)	Odds ratios (95% confidence interval) for COVID-19					
			Crude^†^	*P*-value	Model 1^†‡^	*P*-value	Model 2^†§^	*P*-value
**Alzheimer’s disease**								
AD	309/8,070 (3.8%)	647/32,280 (2.0%)	2.41 (2.05–2.83)	<0.001*	2.17 (1.84–2.55)	<0.001*	2.11 (1.79–2.50)	<0.001*
Non-AD	7,761/8,070 (96.2%)	31,633/32,280 (98.0%)	1		1		1	
**Parkinson’s disease**								
PD	52/8,070 (0.6%)	109/32,280 (0.3%)	1.94 (1.39–2.72)	<0.001*	1.78 (1.27–2.50)	0.001*	1.41 (1.00–2.00)	0.054
Non-PD	8,018/8,070 (99.4%)	32,171/32,280 (99.7%)	1		1		1	

*AD, Alzheimer’s disease; COVID-19, Coronavirus Disease 2019; PD, Parkinson’s disease.*

*Conditional logistic regression model; significance at p < 0.05.

*^†^Model stratified for age, sex and income.*

*^‡^Model 1 was adjusted for Charlson comorbidity index scores, hypertension and diabetes.*

*^§^Model 2 was adjusted for model 1 plus Alzheimer’s disease and Parkinson’s disease.*

The crude and adjusted ORs of the associations of pre-existing AD or PD with severe COVID-19 and COVID-19-related mortality are shown in Tables 3, 4. Patients with a pre-existing diagnosis of AD had significantly higher odds of severe COVID-19 than patients without a pre-existing diagnosis of AD (adjusted OR in model 2 = 2.21, 95% CI = 1.64–2.98, *P* < 0.001). Similarly, compared with patients who had never been diagnosed with PD, patients who had been diagnosed with PD had a 2.89 times higher odds of developing severe COVID-19 (adjusted OR in model 2 = 2.89, 95% CI = 1.56–5.35, *P* = 0.001). Compared with participants who had not been previously diagnosed with AD, the odds of patients who had been diagnosed with AD were 2.07 times higher for COVID-19-related mortality, with statistical significance (adjusted OR in model 2 = 2.07, 95% CI = 1.42–3.02, *P* < 0.001). Patients who had been diagnosed with PD had a 1.54 times higher odds of COVID-19-related mortality than controls, although the estimates did not reach statistical significance (adjusted OR in model 2 = 1.54, 95% CI = 0.75–3.16, *P* = 0.236).

**TABLE 3:** Crude and adjusted odds ratios of Alzheimer’s disease and Parkinson’s disease for morbidity in COVID-19 participants.

Characteristics	Severe participants (exposure/total, %)	Mild participants (exposure/total, %)	Odds ratios (95% confidence interval) for morbidity					
			Crude	*P*-value	Model 1^†^	*P*-value	Model 2^‡^	*P*-value
**Alzheimer’s disease**								
AD	117/569 (20.6%)	192/7,501 (2.6%)	9.85 (7.68–12.64)	<0.001*	2.49 (1.87–3.32)	<0.001*	2.21 (1.64–2.98)	<0.001*
Non-AD	452/569 (79.4%)	7,309/7,501 (97.4%)	1		1			
**Parkinson’s disease**								
PD	27/569 (4.7%)	25/7,501 (0.3%)	14.90 (8.59–25.84)	<0.001*	4.20 (2.34–7.54)	<0.001*	2.89 (1.56–5.35)	0.001*
Non-PD	542/569 (95.3%)	7,476/7,501 (99.7%)	1		1			

*AD, Alzheimer’s disease; COVID-19, Coronavirus Disease 2019; PD, Parkinson’s disease.*

*Unconditional logistic regression model; significance at p < 0.05.

*^†^Model 1 was adjusted for age, sex, income, Charlson comorbidity index scores, hypertension and diabetes.*

*^‡^Model 2 was adjusted for model 1 plus Alzheimer’s disease and Parkinson’s disease.*

**TABLE 4:** Crude and adjusted odds ratios of Alzheimer’s disease and Parkinson’s disease for mortality in COVID-19 participants.

Characteristics	Deceased participants (exposure/total, %)	Surviving participants (exposure/total, %)	Odds ratios (95% confidence interval) for mortality					
			Crude	*P*-value	Model 1^†^	*P*-value	Model 2^‡^	*P*-value
**Alzheimer’s disease**								
AD	86/237 (36.3%)	223/7,833 (2.8%)	19.44 (14.45–26.14)	<0.001*	2.18 (1.52–3.15)	<0.001*	2.07 (1.42–3.02)	<0.001*
Non-AD	151/237 (63.7%)	7,610/7,833 (97.2%)	1		1		1	
**Parkinson’s disease**								
PD	15/237 (6.3%)	37/7,833 (0.5%)	14.25 (7.71–26.34)	<0.001*	2.17 (1.09–4.30)	0.027*	1.54 (0.75–3.16)	0.236
Non-PD	222/237 (93.7%)	7,796/7,833 (99.5%)	1		1		1	

*AD, Alzheimer’s disease; COVID-19, Coronavirus Disease 2019; PD, Parkinson’s disease.*

*Unconditional logistic regression model; significance at p < 0.05.

*^†^Model 1 was adjusted for age, sex, income, Charlson comorbidity index scores, hypertension and diabetes.*

*^‡^Model 2 was adjusted for model 1 plus Alzheimer’s disease and Parkinson’s disease.*

There were no substantial differences in the results of the subgroup analyses, and most subgroup analyses showed that patients with pre-existing diagnoses of AD or PD had higher odds of developing severe COVID-19 and experiencing COVID-19-related mortality (Supplementary Tables 2, 3). The subgroups of patients with PD who were over 50 years of age, men, and did not have diabetes had significantly higher odds of contracting COVID-19 (Supplementary Table 1).

## Discussion

Our results showed that COVID-19 infection was significantly associated with a pre-existing AD diagnosis but not with a pre-existing PD diagnosis. Our findings confirmed that patients with pre-existing AD had higher odds of severe COVID-19 and COVID-19-related mortality. Although the patients with pre-existing PD were found to have higher odds of both severe disease and mortality due to COVID-19, the difference in mortality was not statistically significant.

Patients with AD are significantly more susceptible to infection with SARS-CoV-2 and have higher case-fatality rate because of certain clinical characteristics of AD. The explanation is thought to be that AD and dementia patients may be prone to having a high viral load because most of them are unable to comply with the recommendations to reduce the transmission of COVID-19 issued by the public health authorities due to their overall cognitive decline (Brown et al., 2020). In addition, the majority of the patients with AD and PD tend to live in care facilities where the risk of infection is considered high. Individuals with dementia, including AD, are also more likely than those without dementia to have cardiovascular comorbidities that are risk factors not only for AD but also for symptomatic and severe COVID-19 and are also more likely to have diabetes and pneumonia (Bauer et al., 2014). Additionally, advanced age, which was observed in most AD patients, has been identified as the most influential risk factor for mortality due to COVID-19 (Livingston and Bucher, 2020). Overall, it is unclear whether AD confers direct vulnerability to infection with SARS-CoV-2 or whether these comorbid conditions in AD contribute to an increased risk of poor outcomes of COVID-19. However, in this study, we identified that the pre-existing diagnosis of AD was closely associated with the risk of contracting COVID-19 and developing severe disease after adjusting for age and various comorbidities. Consistent with our results, a number of studies showed that dementia or AD, especially in the advanced stages of the disease, was associated with a higher risk of contracting COVID-19 and developing severe disease (Atkins et al., 2020; Bianchetti et al., 2020; Covino et al., 2020; Williamson et al., 2020; Yu et al., 2021; Zhou et al., 2021). In particular, one cohort study comprising 12,863 UK Biobank community-dwelling individuals more than 65 years old tested for COVID-19 highlighted that all-cause dementia and AD are age-independent risk factors for disease severity and death in COVID-19.

Meanwhile, a growing number of reports have suggested that AD-related neuropathology aggravates COVID-19 complications. First, amyloid fibrils, the pathologic hallmarks of AD, induce the activation of type I interferon (IFN) cytokines, which are innately produced in response to viral infections (Roy et al., 2020). Specifically, IFN plays a significant role in the host response to viral infection, AD pathology and disease severity and therefore might be a potential therapeutic target in both AD and COVID-19 (Naughton et al., 2020). Second, genomic studies recently demonstrated that angiotensin-converting enzyme 2 (ACE2) is the protein to which SARS-CoV-2 binds to gain entry into cells (Lu et al., 2020), and another recent study revealed that the protein expression level of ACE2 was upregulated in the brains of AD patients (Ding et al., 2020). Recent genomic research strongly indicated that ACE2 gene expression is elevated in the brain tissue of AD patients, which may be an important risk factor for COVID-19 transmission in AD patients (Lim et al., 2020). These neuropathological features of AD indicate that AD itself may be a direct cause of the increased susceptibility to contracting COVID-19 and developing severe disease and the consequent adverse clinical outcomes.

Similar to AD, several features of PD, such as respiratory muscle rigidity and impairment of the cough reflex in conjunction with pre-existing dyspnea, may lead to increased severity of COVID-19, particularly with regard to respiratory complications (Van Wamelen et al., 2020). Furthermore, it is conceivable that indirect consequences of the pandemic, such as increased stress levels, self-isolation, and anxiety, as well as prolonged immobility due to lockdown may worsen the outcomes in PD patients with COVID-19 (Prasad et al., 2020). Despite these characteristics, there are currently contradictory results regarding whether PD alone increases susceptibility to contracting COVID-19 and experiencing unfavorable outcomes. A recent large cohort study in Italy reported that the risks of contracting (7.1 vs. 7.6%) and dying from (age-adjusted OR = 0.45, *P* = 0.20) COVID-19 in patients with mild to moderate PD did not differ from those in the general population (Fasano et al., 2020). A community-based case-control study also reported 12 COVID-19 cases among PD patients (8.5%), whose mean age and disease duration (65.5 and 6.3 years, respectively) were similar to those in the non-PD population (Cilia et al., 2020). Conversely, one study by Antonini et al. (2020), which had a small sample size, investigated 10 PD patients and found that older age (mean, 78.3 years) and longer disease duration (mean, 12.7 years) were associated with an increased susceptibility to contracting COVID-19, with a high case-fatality rate of 40%. Meanwhile, a multicenter survey in Tuscany, Italy, reported a higher prevalence of COVID-19 in the PD population (0.9%) than the national average (0.24–0.35%) (Del Prete et al., 2021). Similarly, the results from the UK Biobank study showed that PD patients had a higher risk of SARS-CoV-2 infection (OR = 1.74, 95% CI = 1.34–2.27), but not of mortality, due to COVID-19 (Yu et al., 2021).

There are several neurobiological factors connecting PD and COVID-19. First, the ACE2 receptor, which is the protein to which SARS-CoV-2 binds to gain entry into host cells, is highly expressed in dopaminergic neurons (Antonini et al., 2020). It is possible to assume the penetration of SARS-CoV-2 into the brain through the ACE2 receptors is reduced in PD patients because of degenerative changes in the dopaminergic neurons. Second, alpha-synuclein, which is considered the pathological hallmark of PD, is known to have multiple immunomodulatory functions, such as protection against pro-inflammatory responses (Lesteberg and Beckham, 2019). In addition, innate neuron-specific inhibitors of viral infection in the central nervous system have been identified (Ait Wahmane et al., 2020). Finally, several medications used for the treatment of PD have been found to have antiviral properties. Amantadine derivatives, such as amantadine, bananin, and memantine, might exert antiviral effects by inhibiting viral uncoating within the host cell endosome, blocking the enzyme helicase, or inhibiting ion channel activity (Tipton and Wszolek, 2020). However, it is unclear whether these agents are effective antiviral treatments for COVID-19.

Contrary to the above proposals, our results indicate that a pre-existing diagnosis of PD increases the risk of both contracting COVID-19 and experiencing poor outcomes after adjustment for potential confounders; however, the associations of a pre-existing diagnosis of PD with having a diagnosis of COVID-19 infection and COVID-19-related mortality were not significant. Despite the neurobiological background, the reason that pre-existing PD is associated with severe COVID-19 is because the two diseases have common clinical symptoms. The initial manifestations of COVID-19, such as fatigue, anosmia, flushing, or limb pain, could be masked by the non-motor symptoms of PD; thus, the early detection of COVID-19 in PD patients is somewhat challenging (Hainque and Grabli, 2020). It is plausible that PD patients affected by COVID-19 are likely to experience a severe clinical course that can be attributed to a delayed diagnosis.

Similar to the results of all participants, most subgroups stratified by income level showed that patients with a pre-existing diagnosis of AD or PD had a higher likelihood of contracting COVID-19 and experiencing severe COVID-19 disease. Intriguingly, however, only a subgroup of middle-income PD patients were less likely to die due to COVID-19 (aOR = 0.29, 95% CI = 0.05–1.74, *P* = 0.177). It can be inferred that the number of participants in the subgroups was relatively small.

The strengths of this study that warrant mention include the use of a nationwide dataset, which captured all validated cases of AD and PD and laboratory-confirmed cases of COVID-19 in the entire country. In addition, we adjusted for various comorbidities to minimize confounding. Nevertheless, several limitations should be noted. First, we did not assess the region of residence or residence in a care facility. There might be differences in susceptibility, disease severity or mortality according to region of residence because participants living in rural areas have limited access to the healthcare system. In addition, a considerable number of patients with chronic neurodegenerative diseases live in care facilities in Korea, and such facilities have consistently had clusters of COVID-19 cases. Therefore, further studies adjusting for residence type of the participants, which could influence disease susceptibility, are required to elucidate the contribution of pre-existing AD or PD to contracting COVID-19. Second, because of the inherent limitations associated with the use of insurance claims data, there was no information available on other possible confounding factors, such as genetic risk factors for AD or PD, lifestyle factors, stress, and the use of medications (Kuo et al., 2020; Magusali et al., 2021). We could not rule out the effects of these unmeasured confounders. Moreover, information regarding the disease stage or clinical severity of the neurodegenerative diseases was not available in the claims database, although these factors could be closely correlated to contracting COVID-19 and developing severe disease. In fact, although our result was not statistically significant, PD diagnosis was associated with COVID-19 diagnosis in a cohort study using the UK Biobank database (Tahira et al., 2021). Therefore, additional studies in larger populations with more information are warranted to clarify the correlation of the stage of neurodegenerative disease with the severity of COVID-19. Third, AD patients might have been more likely to have COVID-19 infection because they were older than the control participants. Although we calculated the adjusted conditional logistic regression including age as a factor and conducted additional stratified analyses with subgroups of patients by age (<50 years and ≥50 years old), we cannot exclude this limitation. Fourth, the number of severe COVID-19 cases (*n* = 27) and deaths (*n* = 15) among the PD patients was relatively small and thus unable show statistical significance. Fifth, we determined if AD or PD could be a risk factor for contracting COVID-19 by identifying subjects by outcome status in this case-control design study, but a cohort design, which follows the occurrence of COVID-19 infection in the pre-existing AD/PD group and control group (without pre-existing AD/PD), can be the best way to minimize the selection bias. At this time, it is impossible to obtain the results of a follow-up study design in the present study because access to the Korean NHID-COVID DB is currently limited. Therefore, future comparative studies are needed to establish whether the relationship between AD or PD and COVID-19 susceptibility can be replicated. Finally, we cannot rule out the possibility that individuals who were not tested for SARS-CoV-2 infection, namely, control participants, may have contracted COVID-19. Although we cannot exclude the effect of undiagnosed confirmed cases in the control group, the rate was reported to be extremely low in three antibody titer test surveys in Korea (Apio et al., 2020).

## Conclusion

We found that COVID-19 infection was associated with a pre-existing diagnosis of AD but not with a pre-existing diagnosis of PD. In addition, patients with AD had higher odds of developing severe COVID-19 and experiencing COVID-19-related mortality, and patients with PD had higher odds of developing severe COVID-19.

## Data Availability Statement

The datasets presented in this article are not readily available because the data analyzed in this study is subject to the following licenses/restrictions: The current article used a national sample cohort and does not involve data that can be available. Requests to access the datasets should be directed to https://nhiss.nhis.or.kr/bd/ay/bdaya001iv.do.

## Ethics Statement

The studies involving human participants were reviewed and approved by the Ethics Committee of Hallym University (2020-07-022). The Ethics Committee waived the requirement of written informed consent for participation.

## Author Contributions

JK and IC participated in the interpretation of the data and drafted and revised the manuscript. YK, CM, and DY participated in the data collection and data interpretation. HC designed the study, participated in the data collection and data interpretation, and revised the manuscript. All authors approved the final version of the manuscript for publication.

## Conflict of Interest

The authors declare that the research was conducted in the absence of any commercial or financial relationships that could be construed as a potential conflict of interest.

## Publisher’s Note

All claims expressed in this article are solely those of the authors and do not necessarily represent those of their affiliated organizations, or those of the publisher, the editors and the reviewers. Any product that may be evaluated in this article, or claim that may be made by its manufacturer, is not guaranteed or endorsed by the publisher.
