# Supplementary material for: The Association of Pre-existing Diagnoses of Alzheimer’s Disease and Parkinson’s Disease and Coronavirus Disease 2019 Infection, Severity and Mortality: Results From the Korean National Health Insurance Database
Source: Front Aging Neurosci. 2022 Mar 3;14:821235. doi: 10.3389/fnagi.2022.821235 (PMC8934421; doi:10.3389/fnagi.2022.821235)
Supplement: Supplementary file 1 [file Table_1.DOCX]

**TABLE S1** Stratified subgroup analyses of crude and adjusted odds ratios of the association between preexisting Alzheimer’s disease or Parkinson’s disease and COVID-19 in total participants.

| **Characteristics** | | **COVID-19** | **Control** | **Odds ratios (95% confidence interval) for COVID-19** | | | | | |
| --- | --- | --- | --- | --- | --- | --- | --- | --- | --- |
|  |  | **(exposure/total, %)** | **(exposure/total, %)** | **Crude** | **P value** | **Model 1^†^** | **P value** | **Model 2^‡^** | **P value** |
| Age <50 years old (n = 21,410) | | | |  |  |  |  |  |  |
|  | AD | 2/4,282 (0.0%) | 3/17,128 (0.0%) | 2.67 (0.45-15.96) | 0.283 | 1.99 (0.30-13.05) | 0.476 | 1.99 (0.30-13.04) | 0.476 |
|  | PD | 0/4,282 (0.0%) | 3/17,128 (0.0%) | N/A |  | N/A |  | N/A |  |
| Age ≥50 years old (n = 18,940) | | | |  |  |  |  |  |  |
|  | AD | 307/3,788 (8.1%) | 644/15,152 (4.3%) | 2.41 (2.05-2.83) | <0.001^*^ | 2..19 (1.86-2.58) | <0.001 | 2.13 (1.81-2.52) | <0.001^*^ |
|  | PD | 52/3,788 (1.4%) | 106/15,152 (0.7%) | 2.00 (1.43-2.80) | <0.001^*^ | 1.84 (1.31-2.59) | <0.001^*^ | 1.45 (1.02-2.06) | 0.037^*^ |
| Men (n = 16,180) | | | |  |  |  |  |  |  |
|  | AD | 100/3,236 (3.1%) | 179/12,944 (1.4%) | 2.57 (1.96-3.36) | <0.001^*^ | 2.24 (1.70-2.95) | <0.001^*^ | 2.13 (1.61-2.82) | <0.001^*^ |
|  | PD | 21/3,236 (0.6%) | 33/12,944 (0.3%) | 2.60 (1.49-4.53) | 0.001^*^ | 2.53 (1.45-4.43) | 0.001^*^ | 1.98 (1.11-3.52) | 0.020^*^ |
| Women (n = 24,170) | | | |  |  |  |  |  |  |
|  | AD | 209/4,834 (4.3%) | 468/19,336 (2.4%) | 2.32 (1.90-2.85) | <0.001^*^ | 2.12 (1.73-2.61) | <0.001^*^ | 2.10 (1.71-2.58) | <0.001^*^ |
|  | PD | 31/4,834 (0.6%) | 76/19,336 (0.4%) | 1.66 (1.08-2.53) | 0.020^*^ | 1.48 (0.96-2.27) | 0.075 | 1.18 (0.76-1.83) | 0.464 |
| Low income (n = 15,525) | | | |  |  |  |  |  |  |
|  | AD | 141/3,105 (4.5%) | 290/12,420 (2.3%) | 2.53 (1.98-3.23) | <0.001^*^ | 2.29 (1.78-2.93) | <0.001^*^ | 2.21 (1.72-2.84) | <0.001^*^ |
|  | PD | 22/3,105 (0.7%) | 39/12,420 (0.3%) | 2.30 (1.36-3.92) | 0.002^*^ | 2.12 (1.24-3.63) | 0.006^*^ | 1.61 (0.93-2.79) | 0.093 |
| Middle income (n = 11,735) | | | |  |  |  |  |  |  |
|  | AD | 54/2,347 (2.3%) | 125/9,388 (1.3%) | 2.04 (1.41-2.97) | <0.001^*^ | 1.87 (1.28-2.73) | 0.001^*^ | 1.80 (1.23-2.63) | 0.003^*^ |
|  | PD | 11/2,347 (0.5%) | 19/9,388 (0.2%) | 2.36 (1.11-5.01) | 0.025^*^ | 2.28 (1.07-4.86) | 0.034^*^ | 1.97 (0.91-4.27) | 0.086 |
| High income (n = 13,090) | | | |  |  |  |  |  |  |
|  | AD | 114/2,618 (4.4%) | 232/10,472 (2.2%) | 2.48 (1.90-3.24) | <0.001^*^ | 2.18 (1.66-2.86) | <0.001^*^ | 2.17 (1.64-2.86) | <0.001^*^ |
|  | PD | 19/2,618 (0.7%) | 51/10,472 (0.5%) | 1.51 (0.88-2.58) | 0.132 | 1.34 (0.78-2.31) | 0.291 | 1.06 (0.61-1.86) | 0.828 |
| CCI scores = 0 (n = 36,362) | | | |  |  |  |  |  |  |
|  | AD | 134/6,725 (2.0%) | 403/29,569 (1.4%) | 1.48 (1.21-1.80) | <0.001^*^ | 1.71 (1.40-2.10) | <0.001^*^ | 1.68 (1.36-2.06) | <0.001^*^ |
|  | PD | 23/6,725 (0.3%) | 68/29,569 (0.2%) | 1.49 (0.93-2.40) | 0.097 | 1.65 (1.03-2.66) | 0.039^*^ | 1.38 (0.85-2.24) | 0.193 |
| CCI scores = 1 (n = 2,383) | | | |  |  |  |  |  |  |
|  | AD | 93/869 (10.7%) | 137/1,514 (9.0%) | 1.21 (0.91-1.59) | 0.189 | 1.71 (1.27-2.32) | 0.001^*^ | 1.68 (1.23-2.29) | 0.001^*^ |
|  | PD | 16/869 (1.8%) | 22/1,514 (1.5%) | 1.27 (0.66-2.44) | 0.468 | 1.58 (0.81-3.05) | 0.177 | 1.27 (0.65-2.50) | 0.481 |
| CCI scores ≥2 (n = 1,605) | | | |  |  |  |  |  |  |
|  | AD | 82/476 (17.2%) | 107/1,129 (9.5%) | 1.99 (1.46-2.71) | <0.001^*^ | 1.84 (1.31-2.59) | <0.001^*^ | 1.82 (1.29-2.56) | 0.001^*^ |
|  | PD | 13/476 (2.7%) | 19/1,129 (1.7%) | 1.64 (0.80-3.35) | 0.174 | 1.49 (0.72-3.07) | 0.282 | 1.20 (0.57-2.53) | 0.624 |
| Non-hypertension (n = 35,158) | | | |  |  |  |  |  |  |
|  | AD | 104/6,413 (1.6%) | 220/25,745 (0.9%) | 1.91 (1.51-2.42) | <0.001^*^ | 1.96 (1.54-2.50) | <0.001^*^ | 1.91 (1.49-2.45) | <0.001^*^ |
|  | PD | 20/6,413 (0.3%) | 44/25,745 (0.2%) | 1.83 (1.08-3.11) | 0.025 | 1.81 (1.06-3.08) | 0.029 | 1.39 (0.81-2.41) | 0.235 |
| Hypertension (n = 8,192) | | | |  |  |  |  |  |  |
|  | AD | 205/1,657 (12.4%) | 427/6,535 (6.5%) | 2.02 (1.69-2.41) | <0.001^*^ | 2.30 (1.90-2.79) | <0.001^*^ | 2.24 (1.85-2.72) | <0.001^*^ |
|  | PD | 32/1,657 (1.9%) | 65/6,535 (1.0%) | 1.96 (1.28-3.00) | 0.002^*^ | 2.01 (1.31-3.09) | 0.002^*^ | 1.52 (0.97-2.36) | 0.065 |
| Non-diabetes (n = 35,975) | | | |  |  |  |  |  |  |
|  | AD | 200/7,101 (2.8%) | 447/28,874 (1.5%) | 1.84 (1.56-2.18) | <0.001^*^ | 1.99 (1.67-2.38) | <0.001^*^ | 1.91 (1.59-2.29) | <0.001^*^ |
|  | PD | 37/7,101 (0.5%) | 66/28,874 (0.2%) | 2.29 (1.53-3.42) | <0.001^*^ | 2.36 (1.57-3.53) | <0.001^*^ | 1.82 (1.20-2.77) | 0.005^*^ |
| Diabetes (n = 4,375) | | | |  |  |  |  |  |  |
|  | AD | 109/969 (11.2%) | 200/3,406 (5.9%) | 2.03 (1.59-2.60) | <0.001^*^ | 2.38 (1.83-3.10) | <0.001^*^ | 2.39 (1.83-3.12) | <0.001^*^ |
|  | PD | 15/969 (1.5%) | 43/3,406 (1.3%) | 1.23 (0.68-2.22) | 0.494 | 1.32 (0.73-2.40) | 0.365 | 0.94 (0.51-1.74) | 0.845 |

*AD, Alzheimer’s disease; CCI, Charlson comorbidity index; COVID-19, Coronavirus Disease 2019; N/A,* Not applicable; *PD, Parkinson’s disease.*

^*^ Conditional or unconditional logistic regression model, Significance at p <0.05.

^†^ Model 1 was adjusted for age, sex, income, CCI scores, hypertension and diabetes.

^‡^ Model 2 was adjusted for model 1 plus Alzheimer’s disease and Parkinson’ disease.
