# Supplementary material for: The Association of Pre-existing Diagnoses of Alzheimer’s Disease and Parkinson’s Disease and Coronavirus Disease 2019 Infection, Severity and Mortality: Results From the Korean National Health Insurance Database
Source: Front Aging Neurosci. 2022 Mar 3;14:821235. doi: 10.3389/fnagi.2022.821235 (PMC8934421; doi:10.3389/fnagi.2022.821235)
Supplement: Supplementary file 2 [file Table_2.DOCX]

**TABLE S2** Subgroup analyses of crude and adjusted odds ratios of the associations of preexisting Alzheimer’s disease or Parkinson’s disease with severe COVID-19 in COVID-19 participants.

| **Characteristics** | | **Severe participants** | **Mild participants** | **Odds ratios (95% confidence interval) for morbidity** | | | | | |
| --- | --- | --- | --- | --- | --- | --- | --- | --- | --- |
|  |  | **(exposure/total, %)** | **(exposure/total, %)** | **Crude** | **P value** | **Model 1^†^** | **P value** | **Model 2^‡^** | **P value** |
| Age <50 years old (n = 4,282) | | | |  |  |  |  |  |  |
|  | AD | 0/98 (0.0%) | 2/4,184 (0.0%) | N/A |  | N/A |  | N/A |  |
|  | PD | 0/98 (0.0%) | 0/4,184 (0.0%) | N/A |  | N/A |  | N/A |  |
| Age ≥50 years old (n = 3,788) | | | |  |  |  |  |  |  |
|  | AD | 117/471 (24.8%) | 190/3,317 (5.7%) | 5.44 (4.22-7.02) | <0.001^*^ | 1.93 (1.42-2.63) | <0.001^*^ | 1.72 (1.25-2.36) | 0.001^*^ |
|  | PD | 27/471 (5.7%) | 25/3,317 (0.8%) | 8.01 (4.61-13.92) | <0.001^*^ | 3.61 (1.98-6.60) | <0.001^*^ | 2.87 (1.54-5.37) | 0.001^*^ |
| Men (n = 3,236) | | | |  |  |  |  |  |  |
|  | AD | 46/306 (15.0%) | 54/2,930 (1.8%) | 9.43 (6.24-14.25) | <0.001^*^ | 2.21 (1.39-3.51) | 0.001^*^ | 1.84 (1.13-2.99) | 0.014^*^ |
|  | PD | 14/306 (4.6%) | 7/2,930 (0.2%) | 20.02 (8.02-50.00) | <0.001^*^ | 5.99 (2.23-16.08) | <0.001^*^ | 4.54 (1.60-12.93) | 0.005^*^ |
| Women (n = 4,834) | | | |  |  |  |  |  |  |
|  | AD | 71/263 (27.0%) | 138/4,571 (3.0%) | 11.88 (8.62-16.37) | <0.001^*^ | 3.13 (2.12-4.61) | <0.001^*^ | 2.88 (1.94-4.29) | <0.001^*^ |
|  | PD | 13/263 (4.9%) | 18/4,571 (0.4%) | 13.15 (6.37-27.15) | <0.001^*^ | 3.46 (1.62-7.38) | 0.001^*^ | 2.18 (0.99-4.79) | 0.053 |
| Low income (n = 3,105) | | | |  |  |  |  |  |  |
|  | AD | 50/196 (25.5%) | 91/2,909 (3.1%) | 10.61 (7.23-15.56) | <0.001^*^ | 2.69 (1.72-4.22) | <0.001^*^ | 2.42 (1.52-3.87) | <0.001^*^ |
|  | PD | 10/196 (5.1%) | 12/2,909 (0.4%) | 12.98 (5.54-30.44) | <0.001^*^ | 3.86 (1.54-9.68) | 0.004^*^ | 2.39 (0.90-6.35) | 0.081 |
| Middle income (n = 2,347) | | | |  |  |  |  |  |  |
|  | AD | 23/161 (14.3%) | 31/2,186 (1.4%) | 11.59 (6.58-20.41) | <0.001^*^ | 3.64 (1.87-7.07) | <0.001^*^ | 3.14 (1.56-6.31) | 0.001 |
|  | PD | 7/161 (4.3%) | 4/2,186 (0.2%) | 24.79 (7.18-85.61) | <0.001^*^ | 5.50 (1.45-20.91) | 0.012^*^ | 3.05 (0.73-12.83) | 0.128 |
| High income (n = 2,618) | | | |  |  |  |  |  |  |
|  | AD | 44/212 (20.8%) | 70/2,406 (2.9%) | 8.74 (5.81-13.15) | <0.001^*^ | 1.86 (1.17-2.97) | 0.009^*^ | 1.68 (1.04-2.70) | 0.033^*^ |
|  | PD | 10/212 (4.7%) | 9/2,406 (0.4%) | 13.19 (5.30-32.82) | <0.001^*^ | 3.82 (1.49-9.80) | 0.005^*^ | 3.15 (1.20-8.30) | 0.020^*^ |
| CCI scores = 0 (n = 6,725) | | | |  |  |  |  |  |  |
|  | AD | 49/333 (14.7%) | 85/6,392 (1.3%) | 12.80 (8.83-18.55) | <0.001^*^ | 3.53 (2.32-5.37) | <0.001^*^ | 3.23 (2.09-4.99) | <0.001^*^ |
|  | PD | 10/333 (3.0%) | 13/6,392 (0.2%) | 15.19 (6.61-34.91) | <0.001^*^ | 4.12 (1.72-9.86) | 0.002^*^ | 2.34 (0.90-6.06) | 0.081 |
| CCI scores = 1 (n = 869) | | | |  |  |  |  |  |  |
|  | AD | 31/110 (28.2%) | 62/759 (8.2%) | 4.41 (2.70-7.20) | <0.001^*^ | 1.62 (0.90-2.93) | 0.109 | 1.45 (0.79-2.67) | 0.231 |
|  | PD | 8/110 (7.3%) | 8/759 (1.1%) | 7.36 (2.70-20.05) | <0.001^*^ | 3.00 (1.05-8.57) | 0.040^*^ | 2.60 (0.89-7.65) | 0.082 |
| CCI scores ≥2 (n = 476) | | | |  |  |  |  |  |  |
|  | AD | 37/126 (29.4%) | 45/350 (12.9%) | 2.82 (1.72-4.62) | <0.001^*^ | 1.47 (0.82-2.64) | 0.196 | 1.22 (0.66-2.24) | 0.531 |
|  | PD | 9/126 (7.1%) | 4/350 (1.1%) | 6.65 (2.01-22.00) | 0.002^*^ | 5.07 (1.39-18.46) | 0.014^*^ | 4.53 (1.20-17.18) | 0.026^*^ |
| Non-hypertension (n = 6,413) | | | |  |  |  |  |  |  |
|  | AD | 33/294 (11.2%) | 71/6,119 (1.2%) | 10.77 (7.00-16.58) | <0.001^*^ | 2.72 (1.67-4.41) | <0.001^*^ | 0.88 (0.86-0.89) | <0.001^*^ |
|  | PD | 9/294 (3.1%) | 11/6,119 (0.2%) | 17.54 (7.21-42.66) | <0.001^*^ | 4.03 (1.55-10.47) | 0.004^*^ | NA |  |
| Hypertension (n = 1,657) | | | |  |  |  |  |  |  |
|  | AD | 84/275 (30.5%) | 121/1,382 (8.8%) | 4.58 (3.34-6.29) | <0.001^*^ | 2.16 (1.48-3.16) | <0.001^*^ | 1.94 (1.32-2.87) | 0.001^*^ |
|  | PD | 18/275 (6.5%) | 14/1,382 (1.0%) | 6.84 (3.36-13.94) | <0.001^*^ | 3.93 (1.85-8.34) | <0.001^*^ | 3.08 (1.41-6.69) | 0.005^*^ |
| Non-diabetes (n = 7,101) | | | |  |  |  |  |  |  |
|  | AD | 65/383 (17.0%) | 135/6,718 (2.0%) | 9.97 (7.26-13.68) | <0.001^*^ | 2.42 (1.68-3.48) | <0.001^*^ | 2.06 (1.41-3.02) | <0.001^*^ |
|  | PD | 19/383 (5.0%) | 18/6,718 (0.3%) | 19.43 (10.11-37.34) | <0.001^*^ | 5.26 (2.64-10.49) | <0.001^*^ | 3.84 (1.87-7.91) | <0.001^*^ |
| Diabetes (n = 969) | | | |  |  |  |  |  |  |
|  | AD | 52/186 (28.0%) | 57/783 (7.3%) | 4.94 (3.25-7.51) | <0.001^*^ | 2.48 (1.51-4.08) | <0.001^*^ | 2.38 (1.43-3.98) | 0.001^*^ |
|  | PD | 8/186 (4.3%) | 7/783 (0.9%) | 4.98 (1.78-13.92) | 0.002^*^ | 2.28 (0.76-6.80) | 0.140 | 1.44 (0.46-4.51) | 0.534 |

*AD, Alzheimer’s disease; CCI, Charlson comorbidity index; COVID-19, Coronavirus Disease 2019; N/A,* Not applicable; *PD, Parkinson’s disease.*

^*^ Unconditional logistic regression model, Significance at p <0.05.

^†^ Model 1 was adjusted for age, sex, income, CCI scores, hypertension and diabetes.

^‡^ Model 2 was adjusted for model 1 plus Alzheimer’s disease and Parkinson’ disease.
