# Supplementary material for: The Association of Pre-existing Diagnoses of Alzheimer’s Disease and Parkinson’s Disease and Coronavirus Disease 2019 Infection, Severity and Mortality: Results From the Korean National Health Insurance Database
Source: Front Aging Neurosci. 2022 Mar 3;14:821235. doi: 10.3389/fnagi.2022.821235 (PMC8934421; doi:10.3389/fnagi.2022.821235)
Supplement: Supplementary file 3 [file Table_3.DOCX]

**TABLE S3** Subgroup analyses of crude and adjusted odds ratios of the associations of preexisting Alzheimer’s disease or Parkinson’s disease with COVID-19-related mortality in COVID-19 participants.

| **Characteristics** | | **Dead participants** | **Survived participants** | **Odds ratios (95% confidence interval) for mortality** | | | | | |  |  |  |
| --- | --- | --- | --- | --- | --- | --- | --- | --- | --- | --- | --- | --- |
|  |  | **(exposure/total, %)** | **(exposure/total, %)** | **Crude** | **P value** | **Model 1^†^** | **P value** | **Model 2^‡^** | **P value** |  |  |  |
| Age <50 years old (n = 4,282) | | | |  |  |  |  |  |  |  |  |  |
|  | AD | 0/98 (0.0%) | 2/4,184 (0.0%) | N/A |  | N/A |  | N/A |  |  |  |  |
|  | PD | 0/98 (0.0%) | 0/4,184 (0.0%) | N/A |  | N/A |  | N/A |  |  |  |  |
| Age ≥50 years old (n = 3,788) | | | |  |  |  |  |  |  |  |  |  |
|  | AD | 86/233 (36.9%) | 221/3,555 (6.2%) | 8.83 (6.55-11.90) | <0.001^*^ | 2.18 (1.50-3.16) | <0.001^*^ | 2.07 (1.41-3.03) | <0.001^*^ |  |  |  |
|  | PD | 15/233 (6.4%) | 37/3,555 (1.0%) | 6.55 (3.54-12.11) | <0.001^*^ | 2.16 (1.09-4.29) | 0.028^*^ | 1.55 (0.76-3.17) | 0.233 |  |  |  |
| Men (n = 3,236) | | | |  |  |  |  |  |  |  |  |  |
|  | AD | 35/134 (26.1%) | 65/3,102 (2.1%) | 16.52 (10.46-26.09) | <0.001^*^ | 2.37 (1.38-4.07) | 0.002^*^ | 2.28 (1.29-4.04) | 0.005^*^ |  |  |  |
|  | PD | 7/134 (5.2%) | 14/3,102 (0.5%) | 12.16 (4.82-30.64) | <0.001^*^ | N/A |  | 1.27 (0.40-4.02) | 0.685 |  |  |  |
| Women (n = 4,834) | | | |  |  |  |  |  |  |  |  |  |
|  | AD | 51/103 (49.5%) | 158/4,731 (3.3%) | 28.39 (18.70-43.10) | <0.001^*^ | 1.89 (1.14-3.15) | 0.014^*^ | 1.80 (1.07-3.03) | 0.026^*^ |  |  |  |
|  | PD | 8/103 (7.8%) | 23/4,731 (0.5%) | 17.24 (7.52-39.52) | <0.001^*^ | 2.02 (0.82-4.97) | 0.125 | 1.66 (0.66-4.16) | 0.278 |  |  |  |
| Low income (n = 3,105) | | | |  |  |  |  |  |  |  |  |  |
|  | AD | 40/93 (43.0%) | 101/3,012 (3.4%) | 21.75 (13.79-34.32) | <0.001^*^ | 2.73 (1.55-4.81) | 0.001^*^ | 2.53 (1.41-4.56) | 0.002^*^ |  |  |  |
|  | PD | 7/93 (7.5%) | 15/3,012 (0.5%) | 16.26 (6.47-40.91) | <0.001^*^ | 2.89 (1.00-8.37) | 0.050 | 1.77 (0.58-5.39) | 0.314 |  |  |  |
| Middle income (n = 2,347) | | | |  |  |  |  |  |  |  |  |  |
|  | AD | 19/59 (32.2%) | 35/2,288 (1.5%) | 30.58 (16.12-57.99) | <0.001^*^ | 4.41 (1.77-10.96) | 0.001^*^ | 5.48 (2.09-14.41) | 0.001^*^ |  |  |  |
|  | PD | 3/59 (5.1%) | 8/2,288 (0.3%) | 15.28 (3.95-59.12) | <0.001^*^ | 0.80 (0.17-3.77) | 0.780 | 0.29 (0.05-1.74) | 0.177 |  |  |  |
| High income (n = 2,618) | | | |  |  |  |  |  |  |  |  |  |
|  | AD | 27/85 (31.8%) | 87/2,533 (3.4%) | 13.09 (7.91-21.67) | <0.001^*^ | 1.32 (0.73-2.39) | 0.356 | 1.23 (0.67-2.25) | 0.500 |  |  |  |
|  | PD | 5/85 (5.9%) | 14/2,533 (0.6%) | 11.25 (3.96-31.98) | <0.001^*^ | N/A |  | 2.25 (0.73-6.96) | 0.160 |  |  |  |
| CCI scores = 0 (n = 6,233) | | | |  |  |  |  |  |  |  |  |  |
|  | AD | 37/59 (62.7%) | 97/6,174 (1.6%) | 35.54 (22.75-55.51) | <0.001^*^ | 3.97 (2.27-6.97) | <0.001^*^ | 3.66 (2.05-6.54) | <0.001^*^ |  |  |  |
|  | PD | 7/59 (11.9%) | 16/6,174 (0.3%) | 28.88 (11.63-71.75) | <0.001^*^ | N/A |  | 2.01 (0.67-6.02) | 0.210 |  |  |  |
| CCI scores = 1 (n = 869) | | | |  |  |  |  |  |  |  |  |  |
|  | AD | 21/110 (19.1%) | 72/759 (9.5%) | 8.76 (4.67-16.43) | <0.001^*^ | 1.24 (0.56-2.75) | 0.604 | 1.33 (0.59-2.99) | 0.494 |  |  |  |
|  | PD | 2/110 (1.8%) | 14/759 (1.8%) | 2.63 (0.58-11.92) | 0.211 | 0.54 (0.11-2.72) | 0.459 | 0.49 (0.10-2.53) | 0.396 |  |  |  |
| CCI scores ≥2 (n = 476) | | | |  |  |  |  |  |  |  |  |  |
|  | AD | 28/126 (22.2%) | 54/350 (15.4%) | 3.13 (1.83-5.35) | <0.001^*^ | 1.37 (0.72-2.60) | 0.334 | 1.20 (0.62-2.34) | 0.586 |  |  |  |
|  | PD | 6/126 (4.8%) | 7/350 (2.0%) | 4.23 (1.38-12.93) | 0.011 | 3.02 (0.88-10.39) | 0.079 | 2.75 (0.77-9.86) | 0.121 |  |  |  |
| Non-hypertension (n = 6,413) | | | |  |  |  |  |  |  |  |  |  |
|  | AD | 20/294 (6.8%) | 84/6,119 (1.4%) | 26.12 (15.02-45.40) | <0.001^*^ | 1.97 (1.00-3.90) | 0.052 | 2.12 (1.04-4.35) | 0.040^*^ |  |  |  |
|  | PD | 3/294 (1.0%) | 17/6,119 (0.3%) | 15.07 (4.32-52.52) | <0.001^*^ | N/A |  | 0.63 (0.15-2.74) | 0.539 |  |  |  |
| Hypertension (n = 1,657) | | | |  |  |  |  |  |  |  |  |  |
|  | AD | 20/294 (6.8%) | 84/6,119 (1.4%) | 26.12 (15.02-45.40) | <0.001^*^ | 1.97 (1.00-3.90) | 0.052^*^ | 2.12 (1.04-4.35) | 0.040^*^ |  |  |  |
|  | PD | 3/294 (1.0%) | 17/6,119 (0.3%) | 15.07 (4.32-52.52) | <0.001^*^ | N/A |  | 0.63 (0.15-2.74) | 0.539 |  |  |  |
| Non- diabetes (n = 7,101) | | | |  |  |  |  |  |  |  |  |  |
|  | AD | 44/383 (11.5%) | 156/6,718 (2.3%) | 23.46 (15.74-34.96) | <0.001^*^ | 1.91 (1.18-3.08) | 0.008^*^ | 1.77 (1.08-2.90) | 0.024^*^ |  |  |  |
|  | PD | 9/383 (2.3%) | 28/6,718 (0.4%) | 19.09 (8.81-41.34) | <0.001^*^ | 2.34 (1.01-5.43) | 0.048^*^ | 1.81 (0.75-4.38) | 0.186 |  |  |  |
| Diabetes (n = 969) | | | |  |  |  |  |  |  |  |  |  |
|  | AD | 42/186 (22.6%) | 67/783 (8.6%) | 7.19 (4.55-11.36) | <0.001^*^ | 2.71 (1.54-4.77) | 0.001^*^ | 2.65 (1.48-4.74) | 0.001^*^ |  |  |  |
|  | PD | 6/186 (3.2%) | 9/783 (1.1%) | 5.39 (1.88-15.45) | 0.002^*^ | 1.96 (0.61-6.32) | 0.260 | 1.22 (0.36-4.12) | 0.745 |  |  |  |

*AD, Alzheimer’s disease; CCI, Charlson comorbidity index; COVID-19, Coronavirus Disease 2019; N/A,* Not applicable; *PD, Parkinson’s disease.*

^*^ Unconditional logistic regression model, Significance at p <0.05.

^†^ Model 1 was adjusted for age, sex, income, CCI scores, hypertension and diabetes.

^‡^ Model 2 was adjusted for model 1 plus Alzheimer’s disease and Parkinson’ disease.
